# Error of the method: what is it for?

**DOI:** 10.1590/2176-9451.19.2.025-026.ebo

**Published:** 2014

**Authors:** Rodrigo Hermont Cançado, José Roberto Pereira Lauris

**Affiliations:** 1 Assistant Professor, Program of Professional Master's Degree in Orthodontics, Ingá School; 2 Associate Professor, Department of Pediatric Dentistry, Orthodontics and Collective Health, School of Dentistry of Bauru, University of São Paulo

The aim of this paper is to demonstrate the importance of evaluating the error of the method in Orthodontic scientific studies. Special emphasis will be given to the scientific importance and the different types of the error of the method (systematic and casual), the statistical tests most commonly used to quantify these errors and the clinical meaning of the error of the method for the interpretation of results obtained from orthodontic treatment.

To conduct studies within the Dentistry field, particularly in Orthodontics, the researcher needs to measure continuous quantitative variables, i.e., angles or millimetric distances (for instance, the cephalometric variables of lateral cephalograms and/or variables of study casts). To analyze a lateral cephalogram, cephalometric points are traced and distances and angles are measured. However, it is known that these values will vary between examiners and for the same examiner at different moments. It is difficult to obtain always the same exact value. Thus, most studies do not provide accurate measurements with 100% reliability. Because the variability of measurements, that is, the "errors" will always exist, the magnitude and source of errors are more important than the errors themselves.^2^ In scientific research, measurement without the information on the magnitude of the error makes no sense. Therefore, it is extremely important not only to determine the examiner's accuracy in obtaining measurements, but also to estimate all possible sources of error in a research.

A recently published study identified the 100 most cited articles in the orthodontic literature between 1975 and 2011.^4^ At the top of that list, with 545 citations, is the study conducted by Houston,^3^ which aimed at performing error analysis for the measurements carried out in orthodontic research. This fact highlights the importance of the error of the method for orthodontic research.

The main source of measurement error in Orthodontics is the interference of the examiner in the measurement process. While the examiner does not interfere in the process of measuring a person's weight (because it is enough just to put the individual onto a scale), cephalometry depends on his knowledge and ability to locate the cephalometric points. Ideally, the methods employed should be accurate enough so as to allow their reproduction. When this accuracy is somehow compromised, errors occur. They can be defined as the difference between the value obtained during the process of measurement and the real value of the magnitude of measurement.^5^ These errors, when significant and of great magnitude, affect the reliability of results by increasing or decreasing the real differences among the studied variables. The most common types of errors of scientific methods are the casual and systematic error.^3^

The casual error, also known as random error, occurs due to the difficulty and/or inaccuracy in either identifying or defining certain points. The systematic error, also known as non-random error, occurs when a given measurement is continuously under or super-estimated. If a single examiner makes the measurement, generally, this type of error results from either a modification in the measurement technique or an unconscious bias towards directing the outcomes according to his/her own expectations.^3^ Systematic errors of great magnitude distort the results towards a certain direction, while random errors of great magnitude imply difficulty in reproducing the measurements, leading to question either the validity of the method or the examiner's ability.

To obtain the magnitude of casual and systematic errors, that is, the examiner's accuracy in performing measurements, it is necessary to make new measurements on the material used in agreement with the methods employed. A sufficient number of cases must be measured again; otherwise, only great systematic errors will be identified. Empirically speaking, it is believed that repeating 20% of the measurements is enough; but this is not always the truth. If a study sample comprises 15 patients, repeating the measurements of only three individual is not enough. On the other hand, in a study comprising 500 individuals, it is not necessary to repeat the measurements of 100 patients. The required number partly depends on the standard deviation of the differences between the first and second measurements; however at least 25 cases should be measured again.^3^

Repetition of measurements should be carried out by the same examiner: the so-called intra-examiner error. When measurements are performed by two or more examiners, they must repeat the measurements to calculate the intra-examiner and inter-examiner error, that is, the variability of measurements between examiners. In a scientific research, measurements are taken by two or more examiners for different reasons, but normally, the aim is to demonstrate the reliability and reproducibility of the measurement method.

Systematic errors can be identified by means of many statistical tests, for example, paired t-test, the intraclass correlation, and even the Bland-Altman method. Statistically significant differences indicate the presence of systematic error. A good measurement method should not reveal statistically significant systematic error; however, if we consider that in cephalometry the amount of variables is very large, the significant systematic error is expected in about 5% of the cephalometric variables. For example, if a study comprising 30 cephalometric variables reveals significant systematic error in two of them, this result would be expected, and the method should not be revised.

In cephalometry, one of the methods most commonly used to estimate the magnitude of random errors is that proposed by Dahlberg's formula.^1^ It is not a test of significance, but an evaluation of which is the typical difference between the real measurement and the measurement obtained by the method. Thus, this error is expressed in millimeters or degrees, depending on the measurement unit of the variable. Naturally, measurements involving cephalometric points of difficult visualization and identification show greater casual error. No objective rule states how many millimeters or degrees of casual error would be acceptable, because this depends on the interpretation of the measurement. Many authors claim that errors in linear measurements not greater than 1 mm and angular measurements not greater than 1.5º are acceptable. It is important to remember that the casual error is the estimate of the error when one factor is evaluated. Because these studies comprise samples of many factors, the error of the mean of individuals is smaller than that of the individual error.

In summary, whenever a study involves measurements that depend on the examiner's knowledge and ability, it is important to control the error of the method to demonstrate the reliability and reproducibility of the results obtained - indispensable characteristics of scientific knowledge.
